# Epidemiological Assessment of Imported Coronavirus Disease 2019 (COVID-19) Cases in the Most Affected City Outside of Hubei Province, Wenzhou, China

**DOI:** 10.1001/jamanetworkopen.2020.6785

**Published:** 2020-04-23

**Authors:** Yi Han, Yi Liu, Liyuan Zhou, Enguo Chen, Pengyuan Liu, Xiaoqing Pan, Yan Lu

**Affiliations:** 1 Department of Respiratory Medicine, Sir Run Run Shaw Hospital, Institute of Translational Medicine, Zhejiang University School of Medicine, Hangzhou, Zhejiang, China; 2 Department of Gynecologic Oncology, Women’s Hospital, Institute of Translational Medicine, Zhejiang University School of Medicine, Hangzhou, Zhejiang, China; 3 Department of Mathematics, Shanghai Normal University, Xuhui, Shanghai, China

## Abstract

This decision analytical model describes several key epidemiological features of imported coronavirus disease 2019 (COVID-19) cases in Wenzhou, China.

## Introduction

The coronavirus disease 2019 (COVID-19) outbreak was first identified in Wuhan, the capital of Hubei Province, China, in December 2019.^1^ The disease spread rapidly from Wuhan to other cities. To contain this epidemic, Wuhan was locked down on January 23, 2020. Wenzhou, which has a population of 9.3 million and is located in southeastern China approximately 600 miles from Wuhan, is the most affected Chinese city outside of Hubei. Approximately 48 800 people traveled from Wuhan to Wenzhou from January 10 to 23, 2020. As of February 15, 2020, there were 502 confirmed cases of COVID-19 but no deaths reported in Wenzhou. To stop the spread of COVID-19 in Wenzhou, multiple community containment approaches were implemented beginning January 24, 2020, including quarantine, isolation, traffic control, and social distancing (Figure). This decision analytical model examined several key epidemiological features of imported COVID-19 cases in Wenzhou.

**Figure.**
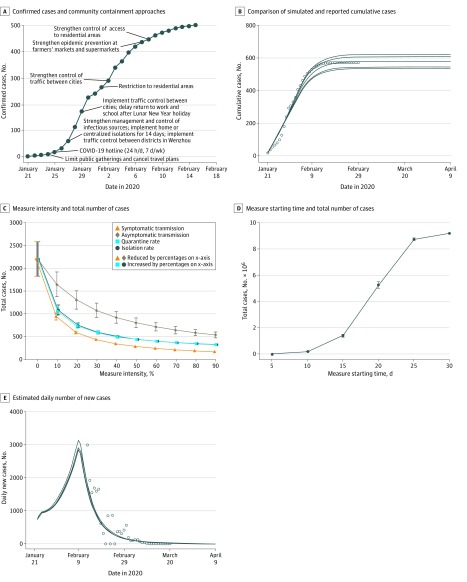
Dynamics of the Spread of Coronavirus Disease 2019 (COVID-19) A, Confirmed cases and community containment approaches taken at different time points in Wenzhou. B, Comparison of simulated (lines) and reported (circles) cumulative cases in Wenzhou. Simulated cases were replicated with 5 different models. C, Measure intensity and total number of cases. In each scenario, 1 measure was changed to 0% to 90% of its baseline value on January 21, 2020, whereas the other 3 parameters remained unchanged. The consequence of changing measure intensity was evaluated by total number of cases 6 months later. D, Measure starting time and total number of cases. The consequence of delayed measure implementation was evaluated by total number of cases 6 months later. E, Estimated daily number of new cases in Wuhan. Simulated cases were replicated with 5 different models and are shown with lines; actual reported cases are shown with circles.

## Methods

 This study was approved by the ethics committees of Sir Run Run Shaw Hospital of Zhejiang University School of Medicine and Women’s Hospital of Zhejiang University School of Medicine, with a waiver of informed consent granted because the data were deidentified. This study follows the Strengthening the Reporting of Observational Studies in Epidemiology (STROBE) reporting guideline.

All confirmed COVID-19 cases reported to the Municipal Health Commission of Wenzhou from January 21 to February 15, 2020, were included in our analysis. Proportions for categorical variables were compared using 2-sided Fisher exact tests with statistical significance set at *P* < .05. The reproduction number *R*_0_ was estimated using the exponential growth method.^2^ The susceptible-asymptomatic-symptomatic-quarantined-isolated-removed model was used to simulate the dynamics of the COVID-19 epidemic (eAppendix in the Supplement).^3^ Data were calculated using the FME package in R statistical software version 1.3.6.1 (R Project for Statistical Computing). Data analysis was conducted from February 2020 to March 2020.

## Results

A total of 482 patients (median age, 48 years; interquartile range, 37-56 years) were included in our analysis. The median age of patients with COVID-19 in Wenzhou who were Wuhan residents was 45 years (interquartile range, 35-53 years), which was 5 years younger than patients who were not Wuhan residents (median, 50 years; interquartile range, 40-61 years) (*P* < .001). Overall, 228 patients (47.3%) were female, and no sex difference was associated with Wuhan residency (81 female patients [43.8%] who were Wuhan residents vs 147 female patients [49.5%] who were not Wuhan residents; *P* = .26). The time between onset of symptoms and diagnosis ranged from 0 to 23 days, with a median of 6 days. Most patients had a fever (317 patients [65.8%]) and cough (232 patients [48.1%]) at disease onset. Other common symptoms at onset included hypodynamia (59 patients [12.2%]), sore throat (50 patients [10.4%]), headache (35 patients [7.3%]), chills (31 patients [6.4%]), and diarrhea (27 patients [5.6%]). Patients who were Wuhan residents were statistically significantly more likely to have chills (18 patients [9.7%] vs 13 patients [4.4%]; *P* = .02) and sore throat (30 patients [16.2%] vs 20 patients [6.7%]; *P* = .001) compared with patients who were not Wuhan residents (Table).

**Table.** Clinical Characteristics of Patients With Coronavirus Disease 2019 in Wenzhou

Characteristic	Patients, No. (%)			*P* value
	Total (N = 482)^a^	Wuhan residence (n = 185)	No Wuhan residence (n = 297)	
Age, median (interquartile range), y	48 (37-56)	45 (35-53)	50 (40-61)	<.001
<20	8 (1.7)	1 (0.5)	7 (2.3)	<.001
20-39	126 (26.1)	61 (33.0)	65 (21.9)	
40-59	258 (53.5)	109 (58.9)	149 (50.2)	
≥60	90 (18.7)	14 (7.6)	76 (25.6)	
Sex				
Female	228 (47.3)	81 (43.8)	147 (49.5)	.26
Male	254 (53.7)	104 (56.2)	150 (50.5)	
Symptoms				
Cough	232 (48.1)	96 (51.9)	136 (45.8)	.16
Fever	317 (65.8)	113 (61.1)	205 (69.0)	.07
Chills	31 (6.4)	18 (9.7)	13 (4.4)	.02
Hypodynamia	59 (12.2)	27 (14.6)	32 (10.8)	.25
Headache	35 (7.3)	17 (9.2)	18 (6.1)	.21
Myalgia	15 (3.1)	5 (2.7)	10 (3.4)	.79
Diarrhea	27 (5.6)	11 (5.9)	16 (5.4)	.84
Dyspnea	13 (2.7)	3 (1.6)	10 (3.4)	.39
Sore throat	50 (10.4)	30 (16.2)	20 (6.7)	.001
Rhinorrhea	14 (2.9)	8 (4.3)	6 (2.0)	.17
Malaise	8 (1.7)	6 (3.2)	2 (0.7)	.06

^a^ Clinical data were not available for 20 of 502 patients.

The basic reproduction number, *R*_0_, of patients with severe acute respiratory syndrome coronavirus 2 was estimated as 2.9 (95% CI, 1.8-4.5) in Wenzhou, which is within the range of those recently reported.^4,5,6^ This indicated that 1 case would produce, on average, 2.9 secondary cases in susceptible populations without any intervention measures.

The susceptible-asymptomatic-symptomatic-quarantined-isolated-removed model was used to simulate the spread of COVID-19 starting on January 21, 2020, in Wenzhou (Figure). Comparison of simulated and reported cumulative cases revealed that our model mimicked the actual spread well. Our estimates suggested that the epidemic would gradually vanish in late February and end in early March after the implementation of community containment approaches in Wenzhou. Then, we used our model to evaluate the impact of 4 different measures on total cumulative number of cases 6 months later. Our simulation showed that the intensity and starting time of control and prevention measures had major impacts on the spread of COVID-19. It is essential to prevent coronavirus transmission between susceptible and infected individuals by quarantine, isolation, and fewer contacts. The total mean (SEM) cumulative number of cases 6 months later would decrease to 440 (16) if the quarantine of infected individuals from the general population before they develop clinical symptoms (ie, asymptomatic individuals) was increased by 50% from the baseline. In contrast, the mean (SEM) number of cumulative cases would increase to 15 576 (1554) if measures were delayed for 5 days after the first diagnosed case. Similarly, the epidemic curves were constructed in Wuhan, suggesting that the COVID-19 epidemic would not stop until early April 2020.

## Discussion

Our epidemic analysis demonstrated that the timely community containment approaches implemented in Wenzhou were associated with the end of the COVID-19 epidemic in early March 2020. These findings suggest that quarantine of asymptomatic individuals is as important as isolation (hospitalization) of infected individuals with symptoms. Therefore, it is recommended that countries with severe epidemics strengthen epidemiological investigations and increase quarantine of close-contact individuals, especially when medical resources are scarce. A limitation of our epidemic analysis is that it is data-driven and limited by its applicability; however, these data have important implications in forecasting and preventing the potential spread of COVID-19 outbreaks in other countries.

## Notes

Supplement. **eAppendix.** Supplemental Methods **eReferences.**
