# Supplementary material for: Epidemiological Assessment of Imported Coronavirus Disease 2019 (COVID-19) Cases in the Most Affected City Outside of Hubei Province, Wenzhou, China
Source: JAMA Netw Open. 2020 Apr 23;3(4):e206785. doi: 10.1001/jamanetworkopen.2020.6785 (PMC7180422; doi:10.1001/jamanetworkopen.2020.6785)
Supplement: Supplement. — eAppendix. Supplemental Methods eReferences. [file jamanetwopen-3-e206785-s001.pdf]

Supplementary Online Content

Han Y, Liu Y, Zhou L, et al. Epidemiological assessment of imported coronavirus disease 2019 (COVID-19) cases in the most affected city outside of Hubei Province, Wenzhou, China. *JAMA Netw Open*. 2020;3(4):e206785. doi:10.1001/jamanetworkopen.2020.6785

eAppendix. Supplemental Methods
eReferences.

This supplementary material has been provided by the authors to give readers additional information about their work.

© 2020 Han Y et al. *JAMA Network Open.*

eAppendix. Supplemental Methods

Statistical analysis of demographic and clinical data

The following patient demographic and clinical data was collected: age, gender, history of Wuhan residency, date of symptom onset, clinical symptoms at onset, and date of diagnosis. Age was described using median and interquartile range (IQR). Mann–Whitney test was used to evaluate the difference in median age between patients from Wuhan or not. Other categorical variables were described as frequencies and percentages. Proportions for categorical variables were examined using Fisher's exact tests or $\chi 2$ tests. P value < 0.05 was considered significant. All statistical analyses were performed using R Statistical Software (version 3.6.2) (https://www.r-project.org/).

Estimating basic reproduction number

The basic reproduction number of SARS-Cov-2 was estimated by the method as described by Wu et al.[1]. Specifically, to adjust the underestimated diagnoses, the reporting rate on the *t*-th day was estimated as

$$\text{Reporting rate (t)} = \frac{1+increase\ fold(t)}{1+\text{max increase fold}} \in (0,1],$$

where the increase fold grew linearly during a period of time, and its growth rate depends on the time that the increase fold started and ended to grow. The adjusted number of daily diagnoses was defined as,

$$\text{a(t)} = \frac{\text{c(t)}}{\text{reporting rate(t)}}$$

where c(t) is the reported number of confirmed cases on the *t*-th day.

We focused on coronavirus confirmed cases who traveled from Wuhan to Wenzhou between January 23 and 27, 2020. The reporting rate was assumed to increase on January 23 and reached the maximum on January 27. The duration time of COVID-19 was consistent with 5 days' incubation of the Severe Acute Respiratory Syndrome (SARS) and the Middle East Respiratory Syndrome (MERS). The reproduction number R0 was estimated using the exponential growth method, implemented in the R0 (version 1.2-6) R package (version 3.6.1)[2]. The serial interval (SI) was modelled as Gamma distribution in which its mean and standard deviation (SD) should be provided [3]. The information of COVID-19 (7.5±3.4) from a recent study was used to set the SI and SD[4].

SEIQJR model

Three features of the susceptible-asymptomatic-symptomatic-quarantined-isolated-removed (SEIQJR) model were suitable to simulate the spread of COVID-19 in the population. First, since both quarantine of asymptomatic individuals exposed to virus and isolation of individuals diagnosed with COVID-19 were used in Wenzhou to control the spread of the epidemic, quarantine (Q) and isolation (J) were considered in our model[5]. Second, asymptomatic infected persons could also be an infection source according to the national's diagnosis and treatment scheme of novel coronavirus pneumonia (The seventh edition). So we considered infectiousness of those individuals at incubation period. Third,

there were asymptomatically infected travelers into the city whom we considered the imported cases. The SEIQJR model could be demonstrated by the following diagram[5],

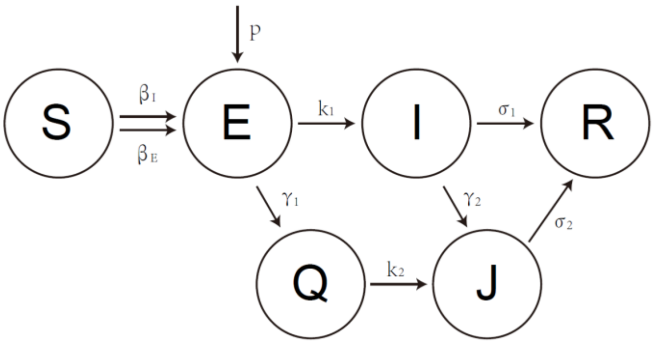

This model can be expressed as,

$$\frac{dS(t)}{dt} = -\frac{S\left(\beta_I I(t) + \beta_E E(t)\right)}{N}$$

$$\frac{dE(t)}{dt} = p + \frac{S\left(\beta_I I(t) + \beta_E E(t)\right)}{N} - (\gamma_1 + k_1)E(t)$$

$$\frac{dI(t)}{dt} = k_1 E(t) - (\gamma_2 + \sigma_1)I(t)$$

$$\frac{dQ(t)}{dt} = \gamma_1 E(t) - k_2 Q(t)$$

$$\frac{dJ(t)}{dt} = k_2 Q(t) + \gamma_2 I(t) - \sigma_2 J(t)$$

$$\frac{dR(t)}{dt} = \sigma_1 I(t) + \sigma_2 J(t)$$

where S(t), E(t), I(t), Q(t), J(t) and R(t) are the number of susceptible, asymptomatic, symptomatic, quarantined, isolated and removed individuals at time t, respectively, so that S(t) + E(t) + I(t) + Q(t) + J(t) + R(t) = N, and N is total population size. Asymptomatic individuals are those who are exposed to the virus, but not yet develop clinical symptoms. Symptomatic individuals are resulted from the development of clinical symptoms of those asymptomatic cases. Quarantined individuals are asymptomatic cases separated from the general population before developing clinical symptoms. Isolation is the separation, such as hospitalization, of infected individuals with symptoms. Removed class includes recovered cases and virus-induced dead individuals. β_I and β_E are transmission coefficients of symptomatic and asymptomatic classes which reflect the effect of control and prevention measures, such as social distancing and reducing contacts, on stopping the spread of virus. γ_1 is quarantine rate of asymptomatic individuals and γ_2 is isolation rate of symptomatic individuals. k_1 and k_2 represent the rate at which asymptomatic and quarantined individuals develop clinical symptoms and thus are transferred to the symptomatic and isolated class. σ_1 and σ_2 are the recovery plus virus-induced death rates of symptomatic and isolated class. p is assumed to be the rate of

© 2020 Han Y et al. *JAMA Network Open.*

imported asymptomatic cases.

k_1 and k_2 were estimated by $1/incubation\_period$.[6] The mean incubation period was used as 5.2 days estimated from a recent study[4]. σ_1 and σ_2 were estimated by $(1-\mu T)/T$ where μ is natural death rate per day and T is expected time in isolation (or expected time until recovery or death)[5]. The annual natural death rate in 2019 was 7.14‰ according to National Bureau of Statistics of China. The median hospitalization time was chosen as 14 and 13 days for σ_1 and σ_2, respectively[7]. Four parameters associated with government policies (β_I, β_E, γ_1 and γ_2) were estimated using a Markov Chain Monte Carlo (MCMC) method with the delayed rejection adaptive Metropolis algorithm and non-informative prior implemented in FME R package (version 1.3.6.1)[8]. The rate of imported cases p was calculated from real data of Wenzhou. Initial values of the susceptible, asymptomatic, symptomatic, quarantined, isolated, and removed individuals on January 21 were respectively estimated as S=9249870, E=76, I=16, Q=36, J=2, and R=0 from real data of Wenzhou. Specifically, as for the number of asymptomatic individuals, because the mean incubation period was nearly 5 days, cases whose onset date was between January 22 and 26 were asymptomatic on January 21. We also assumed those who had exposed history were quarantined at initial time. So the number of asymptomatic individuals was estimated as the number of cases whose onset date was between January 22 and 26 and didn't have exposed history.

The number of removed (recovery and death) and isolated individuals from January 21 to February 8 were used to estimate the policy parameters (β_I, β_E, γ_1, and γ_2) in the SEIQJR model with MCMC. MCMC was repeated for five replicates, and four parameter estimates were used as baseline values. Below are the baseline values:

replicate	β_I	β_E	γ_1	γ_2
1	1.1295	0.1473	0.3297	0.5729
2	1.1762	0.1312	0.5404	0.3232
3	1.1120	0.1451	0.4349	0.4290
4	1.1810	0.1322	0.4356	0.4219
5	1.1700	0.1395	0.5638	0.3074

Wenzhou took a series of measures since January 21. The epidemic curve was simulated starting on January 21, 2020. For simplicity, a two-stage optimization was used for simulating the epidemic dynamic in Wenzhou. In the first stage, measures were taken from January 21 to February 3. The epidemic curve was simulated with the baseline parameter values. In the second stage, stronger measures were taken after February 3. The curve was simulated with 50% reduction in β_I and β_E and 30% increase in γ_1, and γ_2 compared with the baseline values in the first stage. The reported cumulative cases were adjusted with the reporting rate as described above.

We investigated the impact of four different measures on total cumulative number of cases 6 months later. In each scenario, one parameter (measure) was changed to 0-90% of its baseline value whereas the other 3 parameters remained unchanged.

We also investigated the impact of starting time of measures on total cumulative number of cases

© 2020 Han Y et al. *JAMA Network Open.*

6 months later. The measure starting time was the days after diagnosis of the first case. Before the measure starts, the transmission coefficients of symptomatic and asymptomatic classes, β_I and β_E, were set to 1.5 times the baseline values because infected people do nothing to stop transmission. The quarantine rate γ_1 was set to 0 because the asymptomatic individuals would not quarantine themselves. The isolation rate γ_2 was set to 20% of the baseline value because 80% patients experienced mild illness according to the COVID-19 Situation Report-41 from WHO. We assumed that severe patients (20% of cases) would seek medical care themselves. After the measure started, the policy parameters were set to the baseline values.

The SEIQJR model was also used for simulating the epidemic curve starting January 21, 2020 in Wuhan. The baseline values of parameters, β_I, β_E, γ_1, and γ_2, were estimated with hospitalization data from the Municipal Health Commission of Wuhan from January 21 to February 8 using MCMC. Other parameters were estimated with the same methods as Wenzhou. Approximately 48,800 people traveled from Wuhan to Wenzhou before the city-wide shutdown of public transport in Wuhan on January 23. There were 76 asymptomatic and 16 symptomatic infected cases in Wenzhou. There were about 9 million people in Wuhan in January 23 after the city-wide lockdown. Thus, the number of asymptomatic and symptomatic infected cases in Wuhan were estimated as 14,016 and 2951, which were subsequently used as initial values for Wuhan. Other initial values came from reported numbers according to the Municipal Health Commission of Wuhan.

January 23 and February 10 were two important dates for slowing the spread of COVID-19 in Wuhan. Wuhan went on lockdown on January 23 and restriction to residential communities was implemented in Wuhan on February 10. We assumed that the transmission coefficients of symptomatic and asymptomatic classes, β_I and β_E, were reduced to 50% of the baseline values; and quarantine and isolation rates, γ_1 and γ_2, were increased by 50% of the baseline values on January 23. Transmission coefficients were further reduced by 95% and quarantine and isolation rates were increased by 50% on February 10.

eReferences

1. Wu JT, Cowling BJ, Lau EH, et al. School closure and mitigation of pandemic (H1N1) 2009, Hong Kong. *Emerging infectious diseases.* Mar 2010;16(3):538-541.
2. Obadia T, Haneef R, Boelle PY. The R0 package: a toolbox to estimate reproduction numbers for epidemic outbreaks. *BMC Med Inform Decis Mak.* Dec 18 2012;12:147.
3. Vink MA, Bootsma MCJ, Wallinga J. Serial intervals of respiratory infectious diseases: a systematic review and analysis. *American journal of epidemiology.* 2014;180(9):865-875.
4. Li Q, Guan X, Wu P, et al. Early transmission dynamics in Wuhan, China, of novel coronavirus–infected pneumonia. *New England Journal of Medicine.* 2020.
5. Gumel AB, Ruan SG, Day T, et al. Modelling strategies for controlling SARS outbreaks. *P Roy Soc B-Biol Sci.* Nov 7 2004;271(1554):2223-2232.
6. Lipsitch M, Cohen T, Cooper B, et al. Transmission dynamics and control of severe acute respiratory syndrome. *Science.* 2003;300(562):1966-1970.
7. Wang D, Hu B, Hu C, et al. Clinical characteristics of 138 hospitalized patients with 2019 novel coronavirus–infected pneumonia in Wuhan, China. *Jama.* 2020.
8. Soetaert K, Petzoldt T. Inverse Modelling, Sensitivity and Monte Carlo Analysis in R Using Package FME. *Journal of Statistical Software.* Feb 2010;33(3):1-28.

© 2020 Han Y et al. *JAMA Network Open.*
